# Concentration-Dependent N-P Interactions Cause Organ-Specific Responses and Nutrient Allocation in Poplar Seedlings

**DOI:** 10.3390/plants14193037

**Published:** 2025-10-01

**Authors:** Xiaan Tang, Yi Zhang, Changhao Li, Xiaotan Zhi, Chunyan Wang

**Affiliations:** College of Forestry, Northwest A&F University, Xianyang 712100, China; 15078371592@163.com (X.T.); zhangyi.1978@163.com (Y.Z.); 13017532728@163.com (C.L.); 15131648016@163.com (X.Z.)

**Keywords:** N-P interactions, organ-specific responses, concentration-dependence, nutrient uptake, root architecture plasticity

## Abstract

This study explores the complex regulatory mechanisms of nitrogen (N) and phosphorus (P) supply interactions on the growth, root architecture, and nutrient uptake of *Populus* × *euramericana* ‘Neva’ seedlings. It shows that these responses depend on nutrient concentrations and exhibit organ-specific patterns. Low P (0 mM) and sufficient N (15–30 mM) enhances plant height and aboveground biomass by promoting P acquisition processes. At moderate N levels (5–15 mM), P supply is sufficient (0.5–1.5 mM) for root and stem growth. Nitrogen application prioritizes aboveground biomass, reducing the root-to-shoot ratio. Root architecture also responds organ-specifically: sufficient N under low P promotes fine root growth to increase P absorption; under moderate P (0.5 mM), balanced N optimizes branching; and under sufficient P (1.5 mM), N increases root thickness while reducing fine root investment. In terms of P metabolism, moderate N under low P increases P concentrations by upregulating phosphate transporter genes, while sufficient N maintains P use efficiency (PUE). For N metabolism, added P under low N (0 mM) maintains N use efficiency (NUE), while higher N levels (15–30 mM) reduce NUE due to interference in nitrogen transport and enzyme activity. This study highlights the importance of organ-specific resource allocation in adapting to N–P interactions and suggests optimizing fertilization strategies based on soil nutrient status to avoid physiological imbalance.

## 1. Introduction

Nitrogen (N) and phosphorus (P) are indispensable elements for plant growth and development. Plants primarily acquire these nutrients in the form of inorganic phosphate (Pi) and nitrate (NO_3_^−^). However, their availability in soils is often constrained by low bioavailability, limited mobility, and spatial heterogeneity, which collectively impose significant limitations on the productivity of agricultural and forestry ecosystems [1,2,3,4,5].

Traditional studies have mainly focused on the effects of single nutrients. Adequate N supply can promote growth and N use efficiency (NUE) by optimizing root structure (e.g., promoting the growth of superficial roots) and enhancing physiological functions such as photosynthesis [6,7,8]. However, excessive N application may also affect the nutrient functions of the xylem [9,10]. P supply directly drives the growth of both the aboveground and belowground parts of woody plants, biomass accumulation, and P absorption and metabolism [11,12,13], and is regulated by Pi transporter proteins [14]. However, excessive phosphorus supply significantly reduces phosphorus use efficiency (PUE) [15]. Furthermore, N and P absorption and utilization exhibit significant interactions. Co-application can generate synergistic effects: N enhances root-zone phosphatase activity, promoting organic P mineralization and achieving “N-promoted P” [16,17]; conversely, P deficiency exacerbates N limitation [18,19]. The Multiple Limitation Hypothesis (MLH) also supports that under joint limitation, increasing any nutrient can improve growth rates [17,20], indicating that nutrient absorption is a networked regulatory process. Therefore, understanding nutrient signal crosstalk (rather than isolated effects) is crucial for adaptation to heterogeneous environments. In model plants, NO_3_^−^ sensing (e.g., through the NRT1.1B-SPX4 interaction) can regulate P signaling (e.g., *PHR1*) and responsive genes [21,22], while P nutrition also affects genes related to NO_3_^−^ absorption (e.g., *NIGT1*/*NLP*) [23]. NO_3_^−^ signaling (PNR/NSR) itself also coordinates the N-P balance [24,25,26]. However, research on woody plants, especially the response mechanisms of different tissues to N-P interactions, remains scarce.

Nutrient allocation at the organ level is a key strategy by which plants adapt to environmental changes. In plants, leaves typically receive priority in nutrient allocation to support photosynthesis, a fundamental physiological process. The stems primarily function in the transport and temporary storage of materials, while the roots are responsible for nutrient absorption and mechanical support [27,28]. Notably, elements such as N and P are not isolated within different organs but interact significantly. The stoichiometric characteristics of these elements (e.g., C:N:P ratios) also exhibit distinct organ-specific patterns and undergo dynamic adjustments in response to changes in external nutrient supply [29,30,31]. In recent years, increased anthropogenic N input has further disrupted the balance of N and P distribution within plants, leading to changes in nutrient allocation strategies between roots and leaves, which in turn affects the plant’s overall adaptive capacity. This asymmetry in nutrient allocation not only limits plant growth but also has profound negative impacts on ecosystem functions and services [32]. Therefore, understanding the mechanisms of N and P distribution across different plant organs is crucial for maintaining ecosystem stability and optimizing its functions.

Poplar (*Populus* spp.), a fast-growing tree species, often experiences N and P limitations in its growing areas [33,34,35]. To sustain plant growth, fertilizers are frequently applied in large quantities; however, this practice often results in low nutrient use efficiency and environmental issues, including the contamination of surface water and groundwater [36,37,38]. Unfortunately, the physiological and regulatory mechanisms of N-P interactions in various tissues of poplar remain poorly understood. Therefore, this study aims to expose poplar seedlings to 12 different N-P nutrient environments to systematically investigate the effects of varying N and P concentrations on their absorption and metabolism, as well as the tissue-specific differences in these effects. Based on these objectives, we propose the following hypotheses: (1) A significant interactive effect exists between N and P supply: P addition will mitigate the P dilution effect induced by high N and is expected to significantly enhance overall N absorption efficiency; conversely, N addition will enhance P absorption and transport under P stress. (2) Nutrient compartmentalization and organ-specific functions lead to differential responses: we hypothesize that N-P interactions will most significantly impact metabolically active organs (such as leaves), altering their nutrient concentration and stoichiometry, while having a weaker effect on structural organs (such as stems). The roots, as nutrient absorption organs, are expected to exhibit nutrient concentration changes more closely aligned with environmental variations in N and P concentrations.

## 2. Materials and Methods

### 2.1. Plant Materials and Experimental Design

Cuttings (∼10 cm in length, 1 cm in diameter, 1-year-old stems) of the poplar were rooted and subsequently planted in columniform pots with a depth of 20 cm and diameter of 10 cm filled with fine sand, with 3 holes of 0.5 cm diameter at the bottom of the pool for drainage. Plantlets were cultivated in a greenhouse (natural light; day/night temperature: 28/20 °C; relative humidity: 75%).

This experiment utilized a two-factor completely randomized design, incorporating 4 N (N) conditions and 3 phosphorus (P) conditions. Seedlings were irrigated with 20 mL of Hoagland solution every 2 days. After 1 week of cultivation, poplar seedlings exhibiting similar height and growth performance were randomly assigned to different nutrient treatments. The experiment employed 20 mL of modified Hoagland solution, which contained 2 mM MgSO_4_·7H_2_O, 1 mM KCl, 0.5 mM CaSO_4_·2H_2_O, 45 μM H_3_BO_3_, 10 μM MnCl_2_·4H_2_O, 0.8 μM ZnSO_4_·7H_2_O, 0.3 μM CuSO_4_·5H_2_O, 0.4 μM Na_2_MoO_4_·2H_2_O, 20 μM FeSO_4_·7H_2_O, and 20 μM EDTA-2Na, along with varying concentrations of KH_2_PO_4_ and KNO_3_ (mM) (Table 1). This concentration gradient was derived from preliminary experiments and designed to encompass a range of physiological states, from nutrient stress to luxury absorption, to facilitate the observation of significant growth and physiological responses. Potassium (K^+^) concentrations in all treatments were normalized by adding KCl to ensure consistency. Nutrient treatments were applied for 30 d to ensure the acquisition of sufficient plant material for physiological analysis. Afterward, the plants were grown for 2 additional weeks without nutrients, receiving 20 mL of water every 2 d. A total of 180 seedlings were included in the experiment, consisting of 3 N treatments, 4 P treatments, and 15 replicates per treatment. Furthermore, routine maintenance and data measurements were performed in a randomized order to minimize potential systematic errors.

### 2.2. Plant Growth and Dry Matter Production

After the experiment, all 180 seedlings were screened. Plants showing significant disease, pest damage, mechanical injury, or those with measurement values identified as statistical outliers were excluded from the final analysis. From the remaining seedlings, 6 representative individuals were systematically selected from each treatment group. Preference was given to seedlings whose height and stem diameter were closest to the group mean, while extreme individuals with the highest or lowest values were deliberately excluded to ensure that the selected seedlings represented the central tendency of the population. Root morphological assessment was conducted first. Roots were carefully removed from the sand substrate, soaked in water for 30 min to soften adhering particles, and gently rinsed with low-flow water using a soft brush to remove residual sand. The cleaned roots were immediately arranged in a transparent tray containing a thin layer of water (approximately 2–3 mm deep), ensuring the roots were spread out to avoid overlap. Root scanning and analysis were conducted following the protocol outlined by Luo, et al. [39] using the WinRHIZO 2012b analysis system (Regent Instruments Canada Inc., Montreal, QC, Canada), with a resolution of 600 dpi. Subsequently, leaves, stems, and roots of each seedling were rapidly separated on ice for subsampling. Each organ was divided into two subsamples: approximately 40% was accurately weighed, flash-frozen in liquid nitrogen, and stored at −80 °C for enzyme activity assays; the remaining 60% was placed in a DHG-9070A drying oven (Jinghong Instruments, Shanghai, China) at 85 ± 0.5 °C until a constant weight was achieved, defined as a difference of less than 1% between two consecutive measurements.

### 2.3. Assessment of P and N Concentrations

Following the procedure of Wang, et al. [40], the dry powders of seedling roots, stems, and leaves were digested using H_2_SO_4_ and H_2_O_2_. According to the method described by Guo, et al. [41], a continuous flow analyzer (AA3, Bran-Luebbe, Hamburg, Germany) was used to determine N and P concentrations in the digests at 660 nm and 700 nm, respectively. N utilization efficiencies (NUEs) and P utilization efficiencies (PUEs) were calculated using the formula: NUE or PUE = tissue biomass/amount of N or P in the tissue, following the guidelines of Gan, et al. [42].

### 2.4. Determination of Enzymatic Activities

The determination of acid phosphatase (APs, EC 3.1.3.2) activity was carried out following the protocol established by Lei, et al. [43]. The activities of root phosphoenolpyruvate carboxylase (PEPC, EC 4.1.1.31) and malate dehydrogenase (MDH, EC 1.1.1.37) were measured according to the methods of Gajewska, et al. [44] and Lü, et al. [45], respectively. In addition, the activities of glutamine synthetase (GS, EC 6.3.1.2), nitrate reductase (NR, EC 1.7.99.4), glutamate synthase (GOGAT, EC 1.4.7.1), and glutamate dehydrogenase (GDH, EC 1.4.1.2) were systematically assessed based on the method outlined by Luo, et al. [39].

### 2.5. Analysis of the Transcript Levels of Essential Genes Involved in P and N Uptake and Assimilation

The transcriptional levels of 18 genes involved in N and P uptake and assimilation were analyzed by qPCR, following the methods outlined by Li, et al. [46] and Luo, et al. [39]. Fine powder of roots, stems, and leaves (~100 mg) was separately used to isolate total RNA via an RNAprep Pure Plant Plus Kit (DP441, Tiangen Biotech, Beijing, China) with RNase-Free DNase I. After DNA-free treatment, the RNA (∼1 µg) was used to synthesize cDNA with an RT mix with DNase (All-in-one) (R2020S, UE Everbright, Suzhou, China), where the reaction system comprised 20 μL based on the instructions provided by the manufacturer. Quantitative PCR was performed in a 20 µL reaction volume, which contained 10 µL of 2× SYBR Green Master Mix (S2014S, UE Everbright Suzhou, China), 1 µL of cDNA, and 0.2 µL of 10 μM primer, which was explicitly designed based on the target gene’s coding sequence (CDS) for each gene (Table S1) and was developed with Premier 5.0 software (Premier Biosoft, Palo Alto, CA, Canada). Quantitative PCR (qPCR) was conducted using an iQ5 real-time system (Bio-Rad, Hercules, CA, USA). Actin2/7 served as the reference gene. Six independent biological replicates were included, and each PCR reaction was performed in triplicate with a dilution series of the reference gene. The amplification efficiencies of all PCRs ranged from 94% to 107%.

### 2.6. Statistical Analysis

Statistical tests were performed using SPSS (version 23.0; SPSS Inc., Chicago, IL, USA), where the data were tested for normality before the statistical analysis. To examine the effects of N and P treatment and their interactions on experimental variables, all variables were analyzed by two-way ANOVAs using SPSS software. Duncan’s multiple range test was used to conduct pairwise multiple comparisons between treatments (*p* = 0.05). All data were expressed as mean ± standard error (SE). The Cq values obtained after quantitative PCR were normalized, and the fold changes in transcripts were calculated as suggested by Zhu, et al. [47]. Fold changes in transcript levels denoted relative transcript levels of genes compared to reference genes (β-actin), which was calculated by subtracting the reference gene’s quantification cycle (Cq) value from the Cq value of the target gene. The relevant graphs were generated using Origin 2021 (Origin Lab Institute Inc., Northampton, MA, USA).

## 3. Results

### 3.1. Seedling Growth Status and Biomass Production

**Figure 1:**
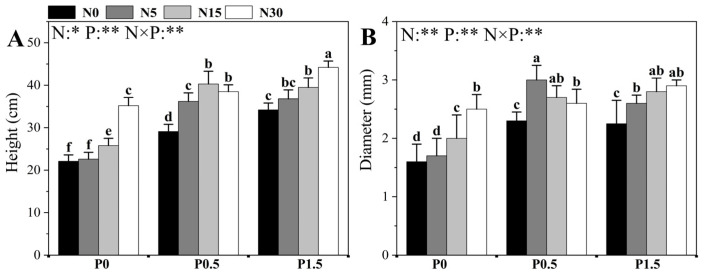
The effects of different N and P treatments on the height (**A**) and ground diameter (**B**) of poplar seedlings. The data are shown as averages ± standard error (SE) with a sample size of 6. Distinct letters atop the bars denote significant differences among treatments (*p* < 0.05). *p*-values obtained from the two-way ANOVAs for N, P and their interactions (N × P) are indicated. * indicates *p* < 0.05; ** indicates *p* < 0.01.

Different nitrogen (N) and phosphorus (P) treatments, as well as their interactions, had significant effects on plant height and diameter of poplar seedlings (Figure 1). The effects of N supply on seedling height and stem diameter were strongly dependent on ambient P conditions. Under low P conditions (P0), when N supply exceeded N5, both plant height and diameter increased significantly with increasing N levels, reaching a peak in the N30 treatment (no significant differences were detected among N0-N5 treatments). At moderate P conditions (P0.5), plant height exhibited a unimodal response to N, peaking at N15 and then plateauing, whereas stem diameter reached its maximum at N5 (significantly greater than under any other N treatment). Under high P conditions (P1.5, adequate P supply), plant height continued to increase with N concentration up to N30, while diameter peaked at N15.

The growth-promoting effects of P addition likewise showed pronounced dependence on ambient N levels. Under low N (N0) and high N (N30, sufficient N supply) conditions, plant height increased linearly with P concentration, achieving the maximum at P1.5. Under low-to-moderate N (N5 and N15) conditions, an increase in P concentration from P0 to P0.5 significantly promoted height growth, but further elevation to P1.5 had no significant effect. The response of diameter to P concentration showed that, except for the N30 treatment, stem diameter significantly increased from P0 to P0.5 at all other N levels (N0, N5, N15), with no significant changes observed from P0.5 to P1.5.

**Figure 2:**
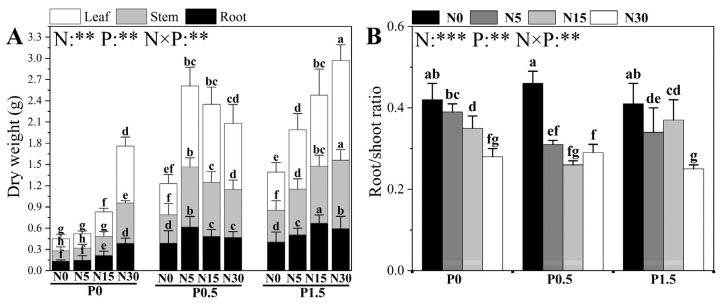
The effects of different N and P treatments on the dry weight of poplar seedlings (**A**) and the root/shoot ratio (**B**). The data are shown as averages ± standard error (SE) with a sample size of 6. Distinct letters atop the bars denote significant differences among treatments (*p* < 0.05). *p*-values obtained from the two-way ANOVAs for N, P and their interactions (N × P) are indicated. ** indicates *p* < 0.01; *** indicates *p* < 0.001.

The different N and P treatments, along with their interactions, significantly affected the dry weight and root/shoot ratio (R/S) of poplar seedlings (Figure 2). The effects of N supply exhibited a pronounced dependence on ambient P conditions. Under P0 conditions, increasing N supply from N0 to N5 had no significant effect on organ dry weights or R/S. However, when N supply exceeded N5, root, stem, and leaf dry weights increased significantly with N concentration, reaching maximum values at N30, while R/S decreased markedly. Under P0.5 conditions, raising N from N0 to N5 significantly increased root, stem, and leaf dry weights and significantly reduced R/S; however, when N supply exceeded N5 (N5 to N30), leaf dry weight and R/S remained unchanged, stem dry weight declined continuously, and root dry weight decreased significantly from N5 to N15 before stabilizing thereafter (N15 to N30). Under P1.5 conditions, stem and leaf dry weights increased steadily with N concentration and peaked at N30, whereas root dry weight reached a maximum at N_15_ and then declined significantly from N15 to N30. R/S decreased significantly from N0 to N5 and from N15 to N30, but showed no significant change from N5 to N15.

The effects of P addition were likewise strongly dependent on ambient N conditions. Under N0, increasing P from P0 to P0.5 significantly increased root, stem, and leaf dry weights while significantly reducing R/S; further elevation to P1.5 produced no significant effect. Under N5, organ dry weights displayed a unimodal pattern, peaking at P0.5 before decreasing, and R/S decreased significantly only from P0 to P0.5. Under N15, the response patterns of root, stem, and leaf dry weights resembled those under N0 conditions (i.e., a significant increase from P0 to P0.5 with no significant change from P0.5 to P1.5), except that root dry weight increased significantly and continuously from P0 to P1.5. Additionally, R/S at P0.5 was significantly lower than at P0 and P1.5. Under N30, root and stem dry weights increased significantly with P concentration from P0 to P1.5, leaf dry weight increased significantly only from P0.5 to P1.5, and R/S decreased significantly from P0.5 to P1.5, with values at P0.5 and P1.5 being significantly lower than at P0.

### 3.2. Root Architecture

**Figure 3:**
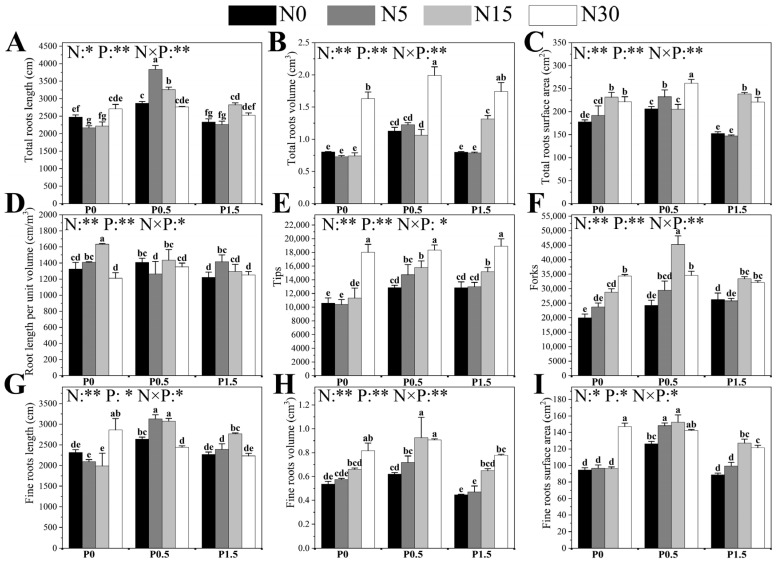
The effects of different N and P treatments on the total root length (**A**), total root volume (**B**), total root surface area (**C**), length per unit volume (**D**), number of root tips (**E**), number of branch points (**F**), fine roots length (0 < root diameter ≤ 0.5 mm) (**G**), fine roots volume (0 < root diameter ≤ 0.5 mm) (**H**) and fine root surface area (0 < root diameter ≤ 0.5 mm) (**I**) of poplar seedlings. The data are shown as averages ± standard error (SE) with a sample size of 6. Distinct letters atop the bars denote significant differences among treatments (*p* < 0.05). *p*-Values obtained from the two-way ANOVAs for N, P and their interactions (N × P) are indicated. * indicates *p* < 0.05; ** indicates *p* < 0.01.

The different N and P treatments, along with their interactions, significantly affected the root system traits (Figure 3 and Table S1). Under P0 conditions, significant increases in total root volume (TRV), tip number (Tips), fine root volume (FRV), fine root surface area (FRS), and fine root length (FRL) were observed only when N was increased from N15 to N30. Fork number (Forks) rose progressively with N input, peaking at N30. Total root surface area (TRS) increased significantly only between N15 and N30, while other N levels had no effect. Total root length (TRL) was highest under N0 and N30 (no difference between them), both exceeding values under N5 and N15 (no difference between the latter two). Length per unit volume (LPV) followed a unimodal pattern, peaking at N15 and declining sharply at N30. At P0.5, TRL, Forks, and FRL exhibited unimodal responses, peaking at N5, N15, and N5, respectively, whereas Tips increased steadily with N supply. Significant increases in TRL occurred only between N15 and N30. TRS showed an increase–decrease–increase trend, reaching its maximum at N30. LPV peaked at N0 and N15 (no difference) and was significantly higher than at N5 and N30. TRV peaked at N15, while FRS showed a similar trend but peaked at N5. At P1.5, TRL, TRS, Forks, FRV, and FRS increased significantly when N rose from N5 to N15. TRV and Tips increased continuously with N, peaking at N30. FRL reached its maximum at N15, exceeding all other N levels. LPV increased from N0 to N5 but declined sharply from N15 to N30 (no differences between N0 and N30, or N5 and N15).

N conditions also strongly influenced P effects on root traits (Figure 3). TRL follows a unimodal curve with increasing P conditions, peaking at P0.5 (except for the N30 treatment). The response patterns of TRV, TRS, FRL, Forks, and FRS to P conditions are similar, though N levels exhibit specific variations: at N15, TRV significantly increases when P conditions are raised from P0 to P0.5, but no difference is observed when P is increased from P0.5 to P1.5. TRS at N15 is unaffected by P conditions. FRL and LPV follow unimodal curves at N0, while at N30, no significant difference is observed when P is increased from P0 to P0.5, but a significant decrease is observed when P increases from P0.5 to P1.5. FRS at N30 follows the same trend (unimodal curves are observed at other N levels). In low N conditions (N0 and N5), Forks increase continuously with increasing P concentrations, peaking at P1.5. Tips increase significantly from P0 to P0.5, and then stabilize (no difference between P0.5 and P1.5), with no effect observed at N30. FRV is regulated by P only at N0 and N15: at N0, increasing P from P0.5 to P1.5 significantly reduces FRV, while at N15, a unimodal curve is observed, with P0.5 reaching the peak. The response of LPV is most complex: at N5, increasing P from P0.5 to P1.5 significantly reduces LPV; at N15, increasing P from P0 to P0.5 significantly decreases LPV, but the effect stabilizes thereafter, with no difference between P0.5 and P1.5.

### 3.3. Plant N, P Uptake

**Figure 4:**
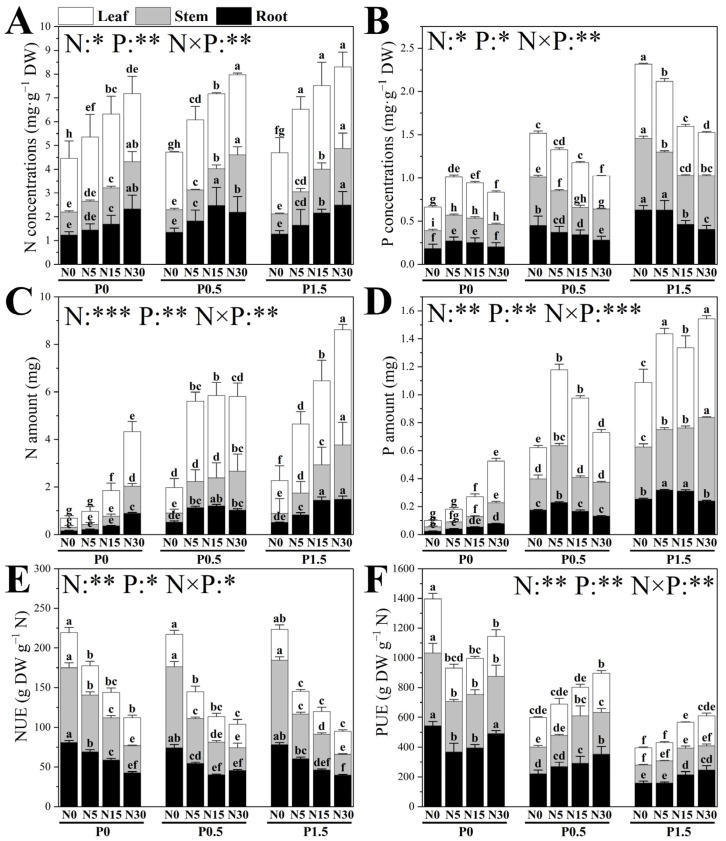
The effects of different N and P treatments on the N concentrations (**A**), P concentrations (**B**), N amount (**C**), P amount (**D**), nitrogen utilization efficiency (NUE) (**E**) and phosphorus utilization efficiency (PUE) (**F**) of poplar seedlings. The data are shown as averages ± standard error (SE) with a sample size of 6. Distinct letters atop the bars denote significant differences among treatments (*p* < 0.05). *p*-values obtained from the two-way ANOVAs for N, P and their interactions (N × P) are indicated. * indicates *p* < 0.05; ** indicates *p* < 0.01; *** indicates *p* < 0.001.

The N and P treatments, along with their interactions, significantly influenced the N concentrations, N amount, N use efficiency (NUE), P concentrations, phosphorus amount, and P use efficiency (PUE) in various parts of poplar seedlings (Figure 4). Under P0, increasing N from N0 to N5 significantly enhanced P concentrations in roots, stems, and leaves, but further N enrichment caused gradual declines in root and leaf P concentrations. In contrast, P amount in all organs rose steadily with N addition, peaking at N30. PUE decreased markedly between N0 and N5, whereas root PUE increased significantly from N15 to N30. At P0.5, P concentrations in roots, stems, and leaves declined progressively with N supply, reaching minima at N30. P amount increased from N0 to N5 but declined thereafter. PUE in all organs increased continuously with higher N levels. Under P1.5, P concentrations in all organs declined with N enrichment, while P amount rose sharply between N0 and N5 and then increased slightly, attaining the highest values at N30. PUE increased consistently with N, also peaking at N30.

P supply also significantly affected N concentrations, amount, and N use efficiency (NUE) in poplar seedling organs (Figure 4). At N0, P addition did not alter root and stem N concentrations but significantly raised leaf N concentrations. Root N amount increased progressively, stem N amount remained unchanged, and leaf N amount rose significantly. NUE in all organs was unaffected by P. At N5, root and stem N concentrations and amounts increased significantly from P0 to P0.5, with no further effect at higher P levels, whereas leaf N concentration and amount rose steadily with P. NUE displayed organ-specific responses: root and stem NUE declined significantly between P0 and P0.5, while leaf NUE decreased between P0.5 and P1.5. At N15, leaf N concentration rose continuously with P enrichment. Root and stem N concentrations increased significantly from P0 to P0.5 but root N concentration declined from P0.5 to P1.5. Root and leaf N amounts rose between P0 and P0.5, with leaf N amount continuing to rise thereafter. Root and stem NUE declined significantly from P0 to P0.5, while leaf NUE remained unchanged. At N30, leaf N concentration increased significantly only between P0 and P0.5, whereas root N concentration rose markedly from P0.5 to P1.5. N amounts of roots, stems, and leaves increased consistently with P addition, reaching maxima at P1.5. NUE declined gradually with P, with a significant drop in leaf NUE between P0 and P0.5.

### 3.4. Enzymatic Activities in N and P Assimilation

**Figure 5:**
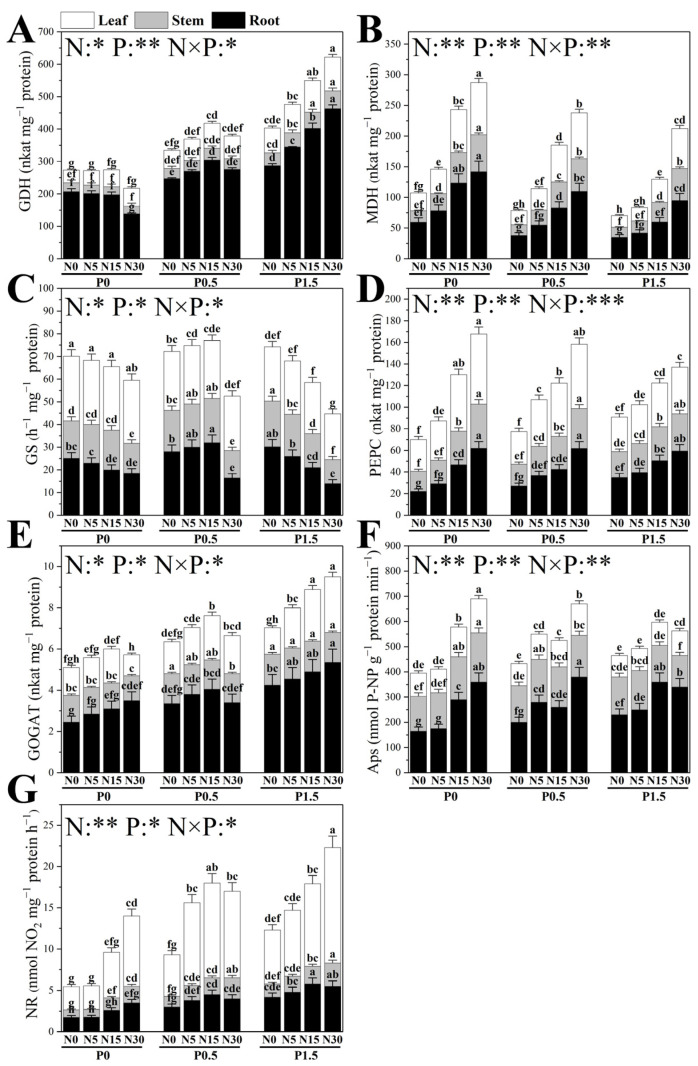
The effects of different N and P treatments on the glutamate dehydrogenase (GDH) (**A**), malate dehydrogenase (MDH) (**B**), glutamine synthetase (GS) (**C**), phosphoenolpyruvate carboxylase (PEPC) (**D**), glutamate synthase (GOGAT) (**E**), acid phosphatases (APs) (**F**) and nitrate reductase (NR) (**G**) of poplar seedlings. The data are shown as averages ± standard error (SE) with a sample size of 6. Distinct letters atop the bars denote significant differences among treatments (*p* < 0.05). *p*-values obtained from the two-way ANOVAs for N, P and their interactions (N × P) are indicated. * indicates *p* < 0.05; ** indicates *p* < 0.01; *** indicates *p* < 0.001.

The application of N and P, along with their interactions, significantly influenced the activity of enzymes related to N and P metabolism in the roots, stems, and leaves of poplar seedlings (Figure 5). N application significantly affected the activity of P metabolism-related enzymes in these tissues, with the magnitude of the effect varying according to environmental P conditions. The activities of malate dehydrogenase (MDH) and phosphoenolpyruvate carboxylase (PEPC) in all organs increased progressively with N supply, peaking at N30. This N-induced stimulation weakened with higher P levels, being strongest under P0, intermediate under P0.5, and weakest under P1.5. For acid phosphatases (APs), activity in roots, stems, and leaves under P0 increased with N only when levels exceeded N5. At P0.5, N enrichment from N0 to N5 significantly increased APs activity in all organs, while further increases from N15 to N30 enhanced APs activity only in roots and leaves. Under P1.5, N from N5 to N15 elevated APs activity solely in roots, whereas N from N15 to N30 reduced APs activity in stems.

N conditions also strongly modulated the effects of P application on N-metabolism–related enzymes (Figure 5). Glutamate dehydrogenase (GDH) activity increased with P across all organs, most prominently in roots, and this stimulation intensified with higher N supply, peaking at N30. Glutamate synthase (GOGAT) and nitrate reductase (NR) showed similar P-induced increases, with the enhancement greatest under N30; for NR, the strongest effect occurred in leaves. Glutamine synthetase (GS) activity responded in a more complex manner. In leaves, GS activity declined with P addition. In stems, GS activity increased with P only under N0; under N5 and N15, it rose significantly from P0 to P0.5, while under N30 it increased from P0.5 to P1.5. In roots, P significantly affected GS activity only at N5 and N15: under N5, activity rose from P0 to P0.5, while under N15 it followed a unimodal trend, peaking at P0.5.

### 3.5. Changes in the Transcript Levels of Essential Genes Involved in P/N Assimilation

**Figure 6:**
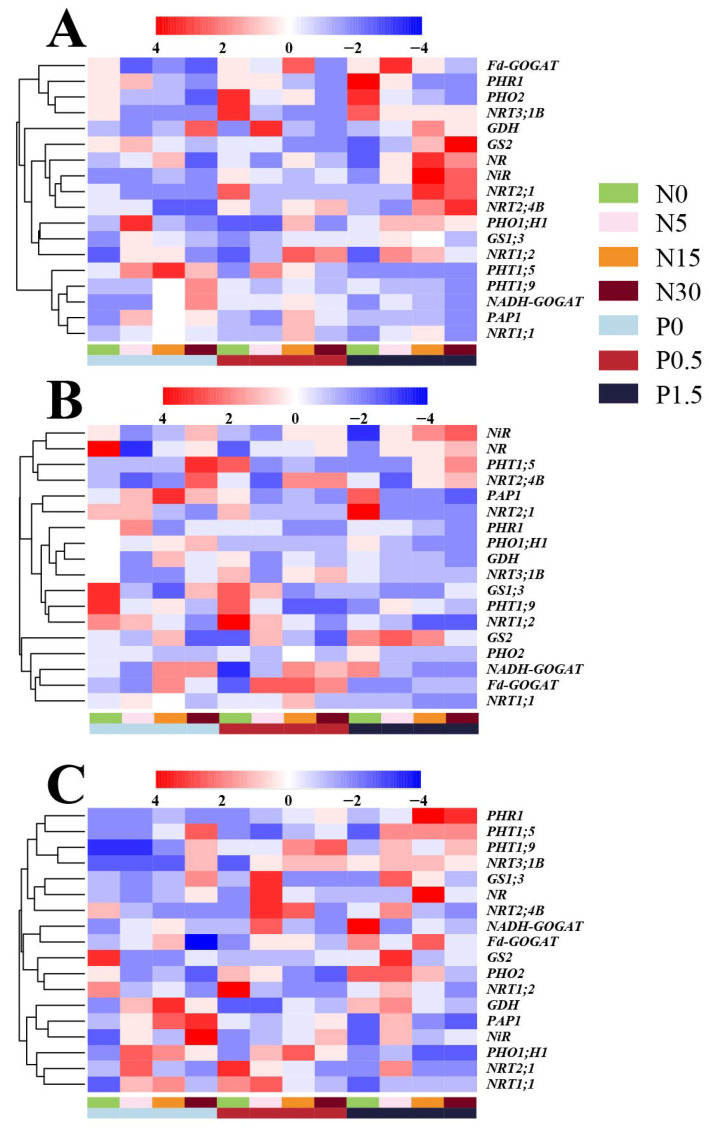
Heatmap showing the transcriptional fold changes in genes involved in the uptake and assimilation of P and N in roots (**A**), stem (**B**), and leaf (**C**) of poplar seedlings. Compared to reference genes, defined as the differences between the Cq value of the target gene and reference gene (β-actin). The color scale indicates fold changes in mRNAs.

Under varying N and P regimes, transcriptional regulation of key genes plays a pivotal role in mediating N–P interactions. N application altered the expression of P transport and metabolism–related genes in a tissue-specific and P-dependent manner (Figure 6). In leaves under P0, transcript levels of *PHT1;5*, *PHT1;9*, and *PAP1* increased monotonically with N supply, peaking at N30, whereas *PHO1;H1* and *PHR1* followed unimodal profiles with maxima at N5 and N15, respectively; *PHO2* declined progressively. Under P0.5, the response patterns became more complex: *PHT1;5* and *PAP1* exhibited U-shaped trends, *PHT1;9* and *PHR1* increased monotonically to N30, *PHO1;H1* showed a single peak at N15, and *PHO2* was negatively regulated. At P1.5, regulation shifted markedly: *PHT1;5* remained positively regulated, while *PHT1;9* and *PAP1* adopted unimodal profiles with peaks advanced to N5; *PHO1;H1* was consistently downregulated, *PHR1* peaked at N15 before a slight decline, and *PHO2* remained strongly repressed. Notably, adequate P supply (P1.5) amplified the N-induced upregulation of *PHR1* and shifted peak positions for *PHT1;9* and related genes. In stems, responses to N were more variable. Under P0, *PHT1;5* transcripts were strongly induced at N30, while *PHO2* and *PHR1* decreased sharply at N15 but rebounded at N30. Under P0.5, *PHT1;5* declined steeply from N0 to N5 and then stabilized; *PHO2* peaked at N15, whereas most other genes were downregulated. At P1.5, *PHT1;5* increased steadily with N, but other genes were broadly repressed, indicating that adequate P supply strengthened N’s positive effect on *PHT1;5* but concurrently suppressed the phosphate starvation response (PSR) pathway. In roots under P0, *PHT1;5* and *PAP1* were upregulated to N15 and then downregulated; *PHO1;H1* peaked earlier at N5; *PHO2* and *PHR1* were consistently repressed. Under P0.5, *PHT1;5* rose sharply at N5 then declined; *PAP1* and *PHO1;H1* exhibited low-amplitude unimodal peaks at N15; *PHO2* and *PHR1* remained downregulated. Under P1.5, N effects were attenuated: *PHT1;5* showed no clear trend, *PAP1* remained stable, *PHO1;H1* fluctuated slightly, and *PHO2* and *PHR1* continued to be suppressed.

Increasing P supply also modulated N metabolism genes in an N-dependent manner across tissues. In leaves under N0, *NRT1;1*, *NRT1;2*, and *NiR* rose then fell (peaking at P0.5); *NADH-GOGAT* and *GS1;3* increased continuously; *GDH* and *NRT2;4B* first decreased then increased. Under N5, *NRT1;2* and *GS2* increased steadily, *NR* rose then fell, and *NRT2;4B* and *Fd-GOGAT* shifted to an increase–decrease pattern. At N15, *NR* was continuously induced, *GDH* was suppressed, *GS2* and *NiR* exhibited unimodal peaks at P0.5, and *NRT1;1* was downregulated. Under N30, *NRT1;2*, *GDH*, and *NiR* were persistently repressed; *NRT3;1B* and *GS1;3* were upregulated only under P1.5; *NRT1;1* and *Fd-GOGAT* followed increase–decrease patterns. In stems under N0, *NRT1;1* and *NRT1;2* rose then fell, *NRT2;4B* increased steadily, *NRT3;1B*, *NiR*, and *GS1;3* declined continuously, and *NRT2;1* increased only under P1.5. *NR*, *GS2*, *Fd-GOGAT*, and *NADH-GOGAT* decreased then increased, whereas *GDH* declined only between P0 and P0.5. Under N5, *NRT1;1*, *NRT1;2*, and *NRT2;1* declined continuously, *NRT2;4B* was unchanged, *NRT3;1B*, *NR*, and *GS2* were steadily induced, and *GS1;3* and *Fd-GOGAT* increased then decreased. *NiR* and *GDH* were induced only from P0.5 to P1.5, and *NADH-GOGAT* only from P0 to P0.5. At N15, *NRT1;1*, *NRT1;2*, and *GDH* were suppressed with increasing P; *NRT2;4B*, *NRT3;1B*, and *GS1;3* peaked then declined; *NRT2;1*, *Fd-GOGAT*, and *NADH-GOGAT* were stable at low P but declined at adequate P. *NiR* rose continuously, *GS2* displayed a V-shaped pattern, and *NR* was stable. Under N30, *NRT1;1*, *NRT1;2*, *NRT3;1B*, and *Fd-GOGAT* rose then fell, while *NRT2;4B*, *NADH-GOGAT*, and *GDH* declined steadily; *NRT2;1* declined only at adequate P; *NR* was unaffected; *NiR* and *GS2* were induced only from P0.5 to P1.5; *GS1;3* decreased only at low P, and *GS2* increased thereafter. In roots under N0, *NRT1;1* was continuously repressed and *GDH* declined then rose; *NRT1;2* decreased then increased, but at N30 this pattern was reversed. Under N5, *NiR* was steadily induced, *GS2* was suppressed, and *GDH* peaked at P0.5 (switching to suppression at N_15_). At N15, *NRT2;4B* and *NR* were upregulated, *NADH-GOGAT* was repressed, and *NiR* induction occurred only from P0.5 to P1.5. At N30, higher P synergistically enhanced expression of most genes: *NiR* and *NRT2;4B* were strongly induced, *NR* gains exceeded those at N15, but *NRT1;1* and *NADH-GOGAT* remained repressed. *GS2* was suppressed by adequate P under N0 but strongly induced under N30. Notably, *NRT1;1* was the only gene downregulated by increasing P at all N levels, whereas *NRT3;1B* increased with P under N5 but switched to a decrease–increase pattern under N30.

## 4. Discussion

### 4.1. Effects of Different N and P Levels on the Growth of Poplar Seedlings

Nutrient limitation in natural soils exerts profound constraints on plant growth and development. While early studies primarily examined the effects of individual nutrients [48,49], recent research has emphasized the complexity and significance of nutrient acquisition dynamics [50,51]. A consistent pattern has emerged whereby increasing nitrogen (N) inputs shift the primary limitation on biomass accumulation from N to phosphorus (P) [52,53]. Excessive N does not necessarily enhance biomass, and may even reduce it—particularly in specific organs—showing strong tissue-specificity [54,55]. These observations point to a complex interplay between N and P nutrition in regulating tree growth. Our findings confirm that the conditions of nitrate (NO_3_^−^) and inorganic phosphate (Pi) shapes one another’s effects on growth, tissue dry mass allocation, and R/S in poplar seedlings. Under low-P conditions, P deficiency may restrict the energy required for N assimilation, leading to a slight N supply that does not significantly affect plant height, stem diameter, or biomass accumulation [56,57,58]. Nevertheless, we observed that sufficient N application significantly improved the above-mentioned growth parameters. This may be because plants require higher N levels to activate P uptake pathways (e.g., by inducing phosphatase activity), thereby promoting the synergistic uptake of P. When P is abundant, the growth-promoting effects of N are fully expressed. Stem diameter and root dry weight peak at lower N levels, while plant height and stem-leaf dry weight require higher N levels. This organ-specific response indicates that the growth-promoting effects of N may primarily support aboveground elongation, while radial growth (stem diameter) and root investment are saturated at lower N levels. As aboveground biomass continues to increase, relative root investment (R/S) significantly decreases. This is consistent with the optimal carbon allocation model, where under sufficient nitrogen, carbon is allocated to photosynthetic organs (leaves) and structural support (stems) to maximize light acquisition [59,60].

P addition also promotes biomass accumulation [61]. However, we found that the effect of P addition is clearly influenced by both environmental conditions and dosage. Under moderate N levels, low P concentrations had a beneficial effect, but further increases in P concentration did not yield significant improvements. This may be because an adequate P supply under medium N levels already meets the plant’s metabolic needs, and excess P could inhibit the uptake of other elements due to ion antagonism (e.g., precipitation with Zn^2+^ or Fe^2+^) [62]. Additionally, under low N conditions, P addition significantly decreased the R/S, suggesting that when P is sufficient, plants reduce root investment and allocate more resources to aboveground parts. Finally, we observed that under sufficient N conditions, P addition continued to promote stem and leaf growth while further reducing the R/S. This response is distinctly different from those observed under low and moderate N conditions. This phenomenon suggests that under sufficient N, the physiological demand for P may increase, such as more P being required for energy transfer in nitrate reduction or additional phospholipid synthesis induced by N for cell division [17,63,64]. Moreover, poplar seedlings demonstrate considerable adaptability to varying N and P applications. Specifically, under optimal N levels, P addition tends to enhance root biomass, thus increasing the R/S. These changes in biomass distribution reflect various adaptive strategies related to nutrient availability, with better nutrition leading to a more stable and equitable biomass distribution, while lower N or P levels favor greater aboveground carbon allocation.

In conclusion, the growth of poplar seedlings is significantly regulated by N and P supply and their interactions, with response patterns showing a strong dependence on nutrient levels. Among the indicators such as plant height, stem diameter, biomass accumulation, and R/S, the regulatory effects of N and P are highly environment-dependent: under low P conditions, sufficient N promotes growth, whereas under sufficient P conditions, moderate N is sufficient to meet the needs. In contrast, the stimulatory effect of P is more pronounced in low or sufficient N environments, while it tends to plateau under moderate N levels.

### 4.2. Effects of Different N and P Levels on Root System Architecture of Poplar Seedlings

Roots are fundamental to plant growth and development, with their morphology and function determining nutrient acquisition efficiency [65,66]. N, an essential macronutrient, regulates root development in a form- and concentration-dependent manner [67]. NO_3_^−^ functions not only as a nutrient but also as a signaling molecule that modulates gene expression and plant development, resulting in root architectural changes [68]. This study demonstrates that environmental P conditions significantly regulate the effect of N supply on the root architecture of poplar seedlings. Under low P conditions, fine root volume (FRV), fine root surface area (FRS), and branching number (Forks) significantly increase only when sufficient N is applied, expanding the root-soil contact interface and providing a direct physical basis for P absorption. Moreover, under these P conditions, only higher N concentrations promote improvements in these indicators, likely due to the need for adequate N to synthesize proteases and cell wall components [69]. Under moderate P conditions, the impact of N addition on root architecture becomes more complex. Total root length (TRL) and fine root length (FRL) peak at lower N levels, while Forks reach their maximum at higher N levels. This gradient response suggests that when basic N requirements are met, carbon may be preferentially allocated to extending root systems to rapidly explore P patches in the soil. However, when N supply is further increased, it may promote root branching complexity to enhance local P acquisition efficiency [70]. Further analysis shows that as N concentration increases, both Forks and TRL increase synchronously, creating a dense root absorption network that efficiently captures N and P nutrients. Under sufficient P conditions, as N supply increases, although total root volume (TRV) and total root surface area (TRS) increase, FRL decreases under sufficient P, and root length volume (LPV) significantly declines. This indicates a shift in the plant’s resource allocation strategy: when P is abundant, plants tend to reduce investment in fine roots with high carbon costs, instead thickening the taproot to enhance mechanical support. However, when N supply is adequate, although thicker roots may facilitate N and P transport by increasing xylem conduit diameter, the significant reduction in fine roots may weaken nutrient absorption capacity. Additionally, the number of root tips (Tips) shows a lack of response to N, further limiting the root system’s ability to detect local nutrients.

The influence of environmental P availability on poplar root structure is a complex ecological process involving multiple biological and chemical mechanisms. Studies have found that under P deficiency, poplar roots exhibit proliferation, which may be an adaptive mechanism to low-P environments [71]. Moreover, P supply levels affect root growth and distribution. For example, under different P concentrations, root length and density significantly increase when P supply is sufficient [72]. Our results indicate that environmental N availability significantly influences the regulation of root architecture by P. P addition generally promotes root growth, but this effect is notably weakened (e.g., no response in branching number) or even reversed under sufficient N conditions, indicating that N abundance weakens the P-promoted root effect. This may result from two main factors: on one hand, plant hormone imbalances induced by sufficient N suppress lateral root development [73,74]; on the other hand, the combined effects of sufficient P and N may increase osmotic stress, reducing root growth [75]. Conversely, under moderate N conditions, as P levels increase, root Forks and FRL reach their peak. This may be because moderate N availability supports P transport and absorption, and sufficient P ensures adequate ATP supply for nitrate reduction, thus providing ample nutrients for root development.

In summary, the root architecture of poplar seedlings shows a clear concentration-dependent interaction in response to N and P supply: under low P conditions, N application activates fine root proliferation, expanding the root-substrate contact interface and enhancing the plant’s ability to absorb P and other nutrients. In contrast, appropriate P application optimizes the balance between branching complexity and elongation, constructing an efficient absorption network that synergistically promotes N capture. Therefore, fertilization strategies should be adjusted according to the substrate P background: low P substrates rely on sufficient N to activate roots, moderate P substrates require balanced ratios to maintain absorption networks, and care should always be taken to avoid root system degradation from excessive N and P fertilization.

### 4.3. Effects of N Application on P Uptake in Poplar Seedlings Under Different P Levels

The effect of N application on P uptake in plants is a complex process involving the interactions between N and P within the plant and the chemical transformations occurring in the soil environment. Studies have shown that N fertilization can enhance the plant’s ability to utilize organic P by inducing an increase in intracellular phosphatase activity [17]. At the molecular level, N conditions has been found to regulate the expression of P metabolism-related genes, thereby influencing the uptake and utilization efficiency of P in plants [23,76]. Notably, soil P conditions have been proven to be a key factor in driving plant trait variation [77,78]. Therefore, potential P limitations in the soil must be considered when implementing ecosystem management and nutrient regulation strategies. Our study indicates that the effect of N fertilization on P metabolism in poplar seedlings exhibits strong dependence on environmental P concentration. Under low P conditions, moderate N application enhanced the activities of APs, MDH, and PEPC and significantly increased P concentrations in roots, stems, and leaves. This is likely a result of the upregulation of P transporter genes [79,80]. However, excessive N application led to a decrease in P concentration in roots and leaves, possibly due to growth dilution effects or feedback inhibition (e.g., *PHO2* upregulation). Given that total P content continued to increase, this suggests that N fertilization promoted biomass accumulation and total P storage [81,82]. In addition, under sufficient N levels, root PUE rebounded, potentially related to enhanced root APs activity and the induction of P recycling genes (*PAP1*) [17,83]. In the medium P treatment, P concentration declined steadily with increasing N application, while total P content exhibited a unimodal trend. PUE in all organs continued to increase, possibly due to enhanced APs activity and the sustained upregulation of P metabolism genes, jointly optimizing P utilization [84,85]. Moreover, we found that increasing P conditions weakened the N-induced enhancement of MDH and PEPC activities, possibly reflecting a negative regulatory effect of P [86,87]. Under sufficient P conditions, P concentration decreased with increasing N levels, but total P content and PUE increased continuously. This might be because N regulation of most P metabolism genes was weakened, prompting plants to shift towards optimizing internal P utilization efficiency [88]. Furthermore, the N-induced enhancement of enzyme activity was markedly attenuated at higher environmental P levels. In summary, under low P conditions, N fertilization primarily promotes P acquisition through initial activation of P uptake and metabolism genes and related enzymes, whereas excessive N shifts the focus towards improving P remobilization efficiency. Under sufficient P conditions, N fertilization mainly promotes biomass accumulation and persistent induction of core P transporters to maximize PUE, while suppressing P stress response pathways to prevent excessive reactions and maintain nutrient balance.

During P nutrition acquisition, different plant organs exhibit distinct response mechanisms. Studies have shown that the acid phosphatase activity of roots increases significantly under P starvation, thereby improving the plant’s ability to utilize organic P [89]. Other studies have indicated that N application can reduce P concentration in leaves, affecting plant growth and physiological function [90]. Furthermore, long-term N addition experiments have found that N addition increases both N and P concentrations in leaves, as well as the leaf N:P ratio, while decreasing N and P concentrations in fine roots [32]. Our results demonstrate that N regulation of P metabolism in poplar seedlings also exhibits pronounced tissue specificity, likely reflecting the functional division of labor among roots, stems, and leaves in P nutrition. As the primary organs for sensing and absorbing nutrients, roots of poplar seedlings prioritize P supply under low P conditions. N fertilization enables roots to maintain sufficient P concentrations, possibly through upregulation of P transporter gene transcription and enhancement of related enzyme activities. Under sufficient N levels, the downregulation of P transporter gene transcription may be the primary cause for decreased P concentration, while sufficient N also promotes P allocation to aboveground parts [88]. In such cases, roots may ensure their PUE by upregulating *PHO2* transcription [91]. As the transport hub, stems display considerable fluctuations in gene expression. Under low P conditions, N fertilization may respond to apical demand by markedly upregulating P transporter genes. In contrast, under sufficient P conditions, N fertilization might lower P metabolic enzyme activities to prioritize P circulation efficiency. This could explain the sharp decline in stem P concentration alongside continual improvement in PUE [92,93,94]. N application during this phase may also reduce P leakage from the xylem by upregulating *PHO2* transcription [91]. Leaves, serving as the systemic regulatory center, integrate N and P signals through mechanisms that may involve the P starvation response (PSR) system [95]. Under low P conditions, increasing N fertilization boosts leaf P content, likely achieved by sustained upregulation of P uptake and metabolism-related gene transcription and associated enzyme activities. Conversely, under sufficient P conditions, N fertilization may promote significant P accumulation in leaves by upregulating the core regulator *PHR1* and inhibiting its negative regulator *PHO2* [91,96,97,98]. In conclusion, poplar seedling roots, stems, and leaves exhibit functionally specialized divisions in P metabolism in response to N regulation: roots under low P prioritize P uptake, while under sufficient N they selectively suppress P uptake genes and enhance PUE; stems dynamically adjust P transporter gene expression and metabolic enzyme activity—strengthening transport capacity under low P and optimizing circulation efficiency under sufficient P; leaves act as the regulatory core, potentially employing the transcription factor PHR1 to modulate downstream genes (such as *PHO2*), activating P uptake and metabolism under low P and markedly enhancing PUE for efficient storage under sufficient P. Ultimately, low N promotes P allocation to roots, while sufficient N drives P enrichment in leaves, forming an integrated whole-plant P optimization strategy dependent on N conditions.

### 4.4. Effect of P Application Under Different N Levels on N Uptake in Poplar Seedlings

N and P are two primary macronutrients essential for plant growth, whose interactions exert profound effects on growth, development, and gene regulation. P conditions not only modulates P uptake but also markedly influences N acquisition and metabolism [99]. For instance, in tea plants, P deficiency substantially alters the expression of genes involved in N metabolism, underscoring P’s pivotal regulatory role [100]. Molecular evidence further indicates that adequate P supply can stimulate the expression of N metabolism-related genes, thereby enhancing N uptake and utilization efficiency [101]. Our results reveal that the influence of P application on N metabolism in poplar seedlings is strongly dependent on ambient N conditions. Under low-N conditions, P addition increased the activities of GDH, GOGAT, and NR in roots and stems, but did not significantly change tissue N concentration or overall NUE. This likely reflects carbon limitation under N scarcity: carbon assimilates may be preferentially invested in biomass production rather than in energy-intensive N assimilation, preventing enhanced enzyme activity from translating into higher NUE [102]. Concurrently, seedlings prioritized N allocation to leaves, as evidenced by upregulated leaf N assimilation genes and increased leaf N content, while N transport and reassimilation in roots and stems were constrained. Elevated GDH activity in root–stem tissues was primarily associated with deamination and ammonia recycling for basal metabolism, rather than with new N assimilation [103]. In addition, proportional increases in biomass and N uptake likely contributed to stable NUE. In contrast, under sufficient N conditions, P application markedly upregulated root N assimilation genes and elevated GDH and NR activities in both roots and leaves, resulting in substantial whole-plant N accumulation. However, NUE declined, potentially due to sustained downregulation of several N uptake and transport genes [104], suppression of GS activity impeding N recycling [105], biomass dilution effects, and increased metabolic energy costs. Collectively, these findings indicate a clear N concentration–dependent pattern: under low N, P application sustains NUE through optimized carbon allocation, organ-level N partitioning toward leaves, and GDH-mediated ammonia recycling; under sufficient N, it stimulates root and leaf N assimilation but limits NUE via reduced N transport, diminished GS activity, and biomass dilution.

Previous studies have demonstrated clear organ-specific responses to N supply, with distinct gene expression patterns in poplar roots and leaves [106], and differential effects on N uptake and utilization efficiencies. N absorbed in summer is largely stored in bulbs to promote leaf growth, whereas autumn assimilation increases leaf N concentration and photosynthetic capacity, enhancing winter survival [107]. Our findings further reveal that P regulation of N metabolism exhibits strong tissue specificity. Roots, as the primary nutrient-sensing and -acquisition organs, respond to P fluctuations via dynamic modulation of N metabolism. Under sufficient N conditions, P application increased root N content, likely through transcriptional upregulation of N metabolism enzymes coupled with elevated enzyme activity [108,109]. Notably, sufficient P also downregulated several nitrate transporter genes, potentially limiting N influx to avoid toxicity and excess energy expenditure—a protective adaptation. Under P sufficiency, plants may preferentially divert resources to P-dependent processes such as energy metabolism and nucleic acid synthesis, rather than to energetically costly N uptake and reduction, explaining the marked decline in root NUE [110,111]. This aligns with observations in Arabidopsis, where N starvation reduces NR activity in both roots and stems [112]. Our data indicate that stems, as relatively inert organs, showed minimal variation in N content across P treatments, likely serving mainly as nutrient transport pathways [113]. In contrast, leaves—as the primary “assimilation factories”—exhibited continuous increases in N concentration and content with P application, attributable to upregulation of N assimilation genes and related enzyme activities. However, in medium- and sufficient-N environments, leaf NUE declined significantly following P addition, likely due to reduced GS activity and downregulation of *NRT1;2* and *GDH* transcripts. Moreover, strong P-induced stimulation of NR activity suggests preferential allocation of carbon skeletons (e.g., photosynthates) toward nitrate reduction rather than ammonium reassimilation via glutamine synthesis [114]. While this strategy supports P-driven primary N assimilation and storage in the short term, lower GS activity restricts ammonium recycling, modestly reducing leaf NUE under P excess. In summary, organ-specific responses to N–P interactions arise from differentiated resource allocation and trade-offs among the three functional modules: roots (acquisition and protection), stems (transport and buffering), and leaves (assimilation and storage). Under low N, poplars adopt a “leaf-prioritized” strategy to maintain photosynthesis; under sufficient N and P, they shift toward a “root-storage” strategy to mitigate stress. This organ-level regulatory divergence represents a key adaptive mechanism for coping with the spatiotemporal heterogeneity of N and P conditions.

## 5. Conclusions

This study elucidates how N and P supply, and their interactions, regulate growth, root system architecture, and nutrient acquisition in poplar seedlings. We demonstrate that N–P interactive effects display marked concentration dependency and organ specificity. Under low-P conditions, sufficient N supply stimulates seedling growth by activating P uptake pathways, including the induction of phosphatases and phosphate transporters. In sufficient-P environments, however, moderate N levels are adequate to sustain growth. Root architectural responses to N–P interactions follow a hierarchical strategy: in low-P substrates, fine-root proliferation relies on sufficient N; under moderate P, balanced N–P supply maintains an efficient absorptive network; under sufficient P, excessive N leads to functional degradation of roots. P metabolism exhibits a “low-P acquisition, sufficient-P optimization” pattern, with roots functioning primarily in stress avoidance and enhancement of PUE, stems maximizing transport efficiency, and leaves serving as storage and regulatory hubs. The degree to which P regulates N metabolism is strongly contingent on environmental N conditions. Under low N, P maintains stable NUE by optimizing carbon allocation and inter-organ N partitioning. Under sufficient N, P enhances assimilation capacity, but NUE ultimately declines due to reduced N transport and suppression of GS activity. These findings deepen our understanding of coordinated N–P adaptation in woody plants and provide a theoretical framework for precision fertilization in plantations—specifically, tailoring N–P ratios to baseline substrate nutrient status to avoid root functional decline and nutrient use inefficiency under combined N–P stress.

While this work provides initial insight into concentration-dependent and organ-specific N–P interactions in poplar seedlings, important gaps remain. Mechanistic resolution is limited, particularly regarding transcriptional regulatory networks within individual organs. The role of mycorrhizal symbiosis and other biotic interactions—critical in natural systems—warrants examination, with emphasis on the interplay between organic acid secretion and mycorrhizal networks under low P. Genetic variation among poplar genotypes in N–P responsiveness also remains unexplored. Furthermore, the integration of carbon metabolism into N–P regulatory frameworks is incomplete; future work should quantify the effects of N–P treatments on photosynthetic carbon allocation, energy metabolism, and nutrient uptake kinetics. Finally, the hormonal signaling pathways mediating organ-specific growth adjustments to N–P regimes require elucidation.

## Figures and Tables

**Table 1:** Experimental treatment design.

Treatment Code	KH_2_PO_4_ (mM)	KNO_3_ (mM)
P0N0	0	0
P0N5	0	5
P0N15	0	15
P0N30	0	30
P0.5N0	0.5	0
P0.5N5	0.5	5
P0.5N15	0.5	15
P0.5N30	0.5	30
P1.5N0	1.5	0
P1.5N5	1.5	5
P1.5N15	1.5	15
P1.5N30	1.5	30

## Data Availability

Relevant data that were generated or analyzed during this study are included in this article. Other data are available upon request to the corresponding author.

## Supplementary Materials

The following supporting information can be downloaded at: https://www.mdpi.com/article/10.3390/plants14193037/s1, Table S1: Primers used for qRT-PCR.
