# Supplementary material for: Concentration-Dependent N-P Interactions Cause Organ-Specific Responses and Nutrient Allocation in Poplar Seedlings
Source: Plants (Basel). 2025 Oct 1;14(19):3037. doi: 10.3390/plants14193037 (PMC12526209; doi:10.3390/plants14193037)
Supplement: Supplementary file 1 [file plants-14-03037-s001.zip › plants-3877402-supplementary.pdf]

Supplementary materials to:

The interaction effects of different nitrogen and phosphorus concentrations in poplars

Xiaan Tang, Chun-Yan Wang[*], Panpan Meng, Guihua Meng, Yingke Yuan, Changhao Li, Xiaotan Zhi
[*]Correspondence: Chun-Yan Wang
E-mail: 15078371592@163.com

Supplementary Table S1. Primers used for qRT-PCR

Gene model or name	Gene name	Closest AGI	Forward Primer（5'-3'）	Reverse Primer（5'-3'）	PCR efficiency
Potri.005G223500	*PHT1;5*	AT2G32830	5‘-TCAACTTCTTCGGCATAGTGTT-3‘	5‘-TGAGGAAGACAGAGCAGGG-3‘	99%
Potri.005G223500	*PHT1;9*	AT1G76430	5‘-ATTGCTCTAGTAAGTTTGGGT-3‘	5‘-CTAGCATTTACAGGATTTGTG-3‘	101%
Potri.010G046300	*PHO1;H1*	AT1G68740	5‘-CTTATTCTCAGGCTTGCTT-3‘	5‘-CCCAACCGACTTCTGT-3‘	107%
Potri.011G052600	*PHO2*	AT2G33770	5‘-TTGACTCTGGAAGCACC-3‘	5‘-TTGGTTCTCACACAGCAT-3‘	105%
Potri.010G158400	*PAP1*	AT1G13750	5‘-GATTCACACCCATCAATACT-3‘	5‘-GCTCAACAGGAAGTTCTCTT-3‘	97%
Potri.002G257800	*PHR1*	AT4G28610	5’-GCAATAACGGAACGGGCAAG-3’	5’-TCAAGCGGTGTCAACTTCCT-3’	95%
Potri.003G111500	*NRT1;1*	AT1G12110	5‘-CTAAACCAAGGGAGGCTCCATGAT-3‘	5‘-CCCAACACAAAAGTAGGCGAAAAG-3‘	102%
Potri.012G070700	*NRT1;2*	AT1G69850	5‘-TCTTTGGTAGCAACTTGAACAA-3‘	5‘-TCTCTCTCTCTCTCGTCTCCCT-3‘	96%
Potri.009G008500	*NRT2;4B*	AT5G60770	5‘-AATAGAGGAAGGGAATGGCTG-3‘	5‘-TGAGGTTGTCCCGAATGATAG-3‘	94%
Potri.015G085000	*NRT3;1B*	AT5G50200	5‘-TCATAGCCTCTTCTTCTACCTTTCC-3‘	5‘-CCACCTTTCAATACTTGTCCG-3‘	103%
Potri.005G172400	*NR*	AT1G37130	5‘-ATCATCGGATCGGAGAGTTGG-3‘	5‘-GACGGTGCTAGTTGGCGTATAG-3‘	102%
Potri.004G140800	*NiR*	AT2G15620	5‘-ACAAGTTGCCGATATTGGGTTCAT-3‘	5‘-CCTCTATCACCCGTCGTAGTCCTG-3‘	101%
Potri.017G131100	*GS1;3*	AT5G37600	5‘-GGCGCAGACCAAGCTTCTC-3‘	5‘-GCGAAGTGACAGATTTAGGATTGC-3‘	105%
Potri.010G029100	*GS2*	AT5G35630	5‘-ATCAGGTGGGTCCCAGTGTG-3‘	5‘-GCGAAGTGACAGATTTAGGATTGC-3‘	103%
Potri.016G036900	*Fd-GOGAT*	AT5G04140	5‘-AACCCAAAGGCATCAGACTCAG -3‘	5‘-AGTAAAGCAGGTCCATCCCAAG-3‘	96%
Potri.012G011700	*NADH-GOGAT*	AT5G53460	5‘-GGTGTTGTGGATATTCCTCCTG-3‘	5‘-TCAGATGCGGCGACAACCC-3‘	97%
Potri.013G058300	*GDH*	AT5G18170	5‘-ATTTATGCTAACTCAGGAGGCGTT-3‘	5‘-GAGGAGGAAACTAGGGCAATACAT-3‘	94%
Potri.009G008500	*NRT2;1*	AT1G08090	5‘-CGGCAGGTATCATGGA-3‘	5‘-ACGGTTTGGTGTGGTACTTG-3‘	102%
	Action 2/7		5‘-CCCATTGAGCACGGTATTGT-3‘	5‘-TACGACCACTGGCATACAGG-3‘	97%
